# Human cancer cells express Slug-based epithelial-mesenchymal transition gene expression signature obtained *in vivo*

**DOI:** 10.1186/1471-2407-11-529

**Published:** 2011-12-30

**Authors:** Dimitris Anastassiou, Viktoria Rumjantseva, Weiyi Cheng, Jianzhong Huang, Peter D Canoll, Darrell J Yamashiro, Jessica J Kandel

**Affiliations:** 1 Institute for Cancer Genetics, Columbia University, New York, NY, USA; 2 Center for Computational Biology and Bioinformatics, Columbia University, New York, NY, USA; 3 Department of Pathology and Cell Biology, Columbia University, New York, NY, USA; 4 Department of Surgery, Columbia University, New York, NY, USA; 5 Department of Pediatrics, Columbia University, New York, NY, USA; 6 Department of Electrical Engineering, Columbia University, New York, NY, USA

**Keywords:** Epithelial-mesenchymal transition, Cancer stem cells, Cancer invasiveness

## Abstract

**Background:**

The biological mechanisms underlying cancer cell motility and invasiveness remain unclear, although it has been hypothesized that they involve some type of epithelial-mesenchymal transition (EMT).

**Methods:**

We used xenograft models of human cancer cells in immunocompromised mice, profiling the harvested tumors separately with species-specific probes and computationally analyzing the results.

**Results:**

Here we show that human cancer cells express in vivo a precise multi-cancer invasion-associated gene expression signature that prominently includes many EMT markers, among them the transcription factor Slug, fibronectin, and α-SMA. We found that human, but not mouse, cells express the signature and Slug is the only upregulated EMT-inducing transcription factor. The signature is also present in samples from many publicly available cancer gene expression datasets, suggesting that it is produced by the cancer cells themselves in multiple cancer types, including nonepithelial cancers such as neuroblastoma. Furthermore, we found that the presence of the signature in human xenografted cells was associated with a downregulation of adipocyte markers in the mouse tissue adjacent to the invasive tumor, suggesting that the signature is triggered by contextual microenvironmental interactions when the cancer cells encounter adipocytes, as previously reported.

**Conclusions:**

The known, precise and consistent gene composition of this cancer mesenchymal transition signature, particularly when combined with simultaneous analysis of the adjacent microenvironment, provides unique opportunities for shedding light on the underlying mechanisms of cancer invasiveness as well as identifying potential diagnostic markers and targets for metastasis-inhibiting therapeutics.

## Background

It has been hypothesized [1-3] that cancer cells become invasive and migratory by undergoing some type of epithelial-mesenchymal transition (EMT) reactivating early embryogenesis programs. Each type of EMT is assumed to be orchestrated by different unknown combinations of multiple interacting transcription factors and signaling pathways. A set of genes comprising an "EMT core signature" was recently derived [4] after triggering EMTs in different ways and observing the resulting shared changes in gene expression. However, the details of the specific types of EMT associated with cancer invasion remain elusive and complicated by the difficulty of detecting signs of EMT in human cancer cells, and the topic remains controversial.

A multi-cancer gene expression signature involving a large set of genes, several among them being EMT markers coordinately overexpressed only in a subset of malignant samples that have exceeded a particular staging threshold specific to each cancer type was recently identified [5]. The signature is present in numerous publicly available datasets from multiple cancer types from all solid tumor types that we tried, including nonepithelial cancers such as neuroblastoma and Ewing's sarcoma but not in leukemia. Examples of heat maps are shown for breast [see Additional file 1], colon [see Additional file 2], Ewing's sarcoma [see Additional file 3], lung [see Additional file 4], ovarian [see Additional file 5], neuroblastoma [see Additional file 6] and leukemia [see Additional file 7], demonstrating the remarkable co-expression of the genes in solid tumors. Among the overexpressed genes are the EMT inducing transcription factor Slug (SNAI2), various collagens and proteinases, α-SMA, fibronectin, fibroblast activation protein, and many extracellular matrix glycoproteins, suggesting a fibroblastic nature after passing through a Slug-based EMT.

The signature, however, has its own special characteristics, one of which is that the strongly co-expressed genes *COL11A1*, *THBS2 *and *INHBA *have a prominent presence. Collagen *COL11A1 *was found to be a reliable proxy for the signature: in each solid tumor dataset that we tried, the list of genes whose expression is most highly correlated (using measures such as mutual information [6] or Pearson correlation) with that of *COL11A1 *consistently includes the other genes of the signature at the top, with the only exception of glioblastoma in which *COL11A1 *is not as prominently co-expressed with the other genes, though the signature is still present otherwise. Notably, E cadherin (*CDH1*) is not downregulated at least at the mRNA level. Furthermore, the signature contains numerous other EMT associated genes. Table 1 identifies the known EMT markers among the list of the 64 genes corresponding to the 100 most overexpressed probe sets previously reported [5], of the signature. Highlighted by bold typeface among these 64 are 20 known EMT-associated genes, 17 coming from the list of 91 upregulated "EMT core signature" [4] genes (*COL5A2*, *FAP*, *POSTN*, *COL1A2*, *COL3A1*, *FBN1*, *TNFAIP6*, *MMP2*, *GREM1*, *BGN*, *CDH11*, *SPOCK1*, *DCN*, *COPZ2*, *THY1*, *PCOLCE*, *PRRX1*) plus the obvious EMT markers *SNAI2*, *FN1*, *ACTA2*. Four additional genes (underlined but not bold in Table 1), *PDGFRB*, *SPARC*, *INHBA*, *COL6A2*, have also been reported as EMT-related [7], for a total of 24 EMT factors. Even without including these additional seven genes, the *P *value of encountering 17 out of 64 genes taken from the list of the 91 EMT core signature genes is 2 × 10^-22^. Therefore, this fibroblastic signature is the result, at least in part, of an EMT.

**Table 1:** Top genes overexpressed in the fibroblastic signature (see text for designation with boldface and underline)

Rank	Probe set	Gene	Rank	Probe set	Gene
1	37892_at	COL11A1	33	202998_s_at	LOXL2

2	203083_at	THBS2	34	201438_at	COL6A3

3	217428_s_at	COL10A1	35	209596_at	MXRA5

4	221729_at	**COL5A2**	36	213764_s_at	MFAP5

5	210511_s_at	INHBA	37	204589_at	NUAK1

6	213909_at	LRRC15	38	217762_s_at	RAB31

7	212488_at	COL5A1	39	201150_s_at	TIMP3

8	204619_s_at	VCAN	40	221541_at	CRISPLD2

9	209955_s_at	**FAP**	41	205422_s_at	ITGBL1

10	202311_s_at	COL1A1	42	207173_x_at	**CDH11**

11	203878_s_at	MMP11	43	213338_at	TMEM158

12	210809_s_at	**POSTN**	44	202363_at	**SPOCK1**

13	202404_s_at	**COL1A2**	45	204051_s_at	SFRP4

14	202952_s_at	ADAM12	46	202283_at	SERPINF1

15	215076_s_at	**COL3A1**	47	209335_at	**DCN**

16	215446_s_at	LOX	48	219655_at	C7orf10

17	210495_x_at	**FN1**	49	219561_at	**COPZ2**

18	201792_at	AEBP1	50	219773_at	NOX4

19	212353_at	SULF1	51	204464_s_at	EDNRA

20	202766_s_at	**FBN1**	52	200974_at	**ACTA2**

21	219087_at	ASPN	53	202273_at	PDGFRB

22	200665_s_at	SPARC	54	61734_at	RCN3

23	202450_s_at	CTSK	55	213139_at	**SNAI2**

24	206026_s_at	**TNFAIP6**	56	220988_s_at	C1QTNF3

25	222020_s_at	HNT	57	205713_s_at	COMP

26	206439_at	EPYC	58	201105_at	LGALS1

27	201069_at	**MMP2**	59	213869_x_at	**THY1**

28	205479_s_at	PLAU	60	202465_at	**PCOLCE**

29	218469_at	**GREM1**	61	209156_s_at	COL6A2

30	201261_x_at	**BGN**	62	221447_s_at	GLT8D2

31	213125_at	OLFML2B	63	204114_at	NID2

32	201744_s_at	LUM	64	205991_s_at	**PRRX1**

The initiation of significant signature overexpression after reaching a particular cancer-type-specific stage suggests an underlying biological mechanism associated with cancer invasion. Given the heterogeneity of cells in tumors, it could also reflect the superposition of several mechanisms. Among other possibilities, the fibroblast-like cells producing the signature could be derived from multiple sources, such as the bone marrow, the local stroma, or the cancer cells after undergoing a mesenchymal transition. A fundamental question, therefore, is which among the genes of the signature are expressed by the cancer cells and which are expressed by the adjacent microenvironment.

We also observed that there is a striking similarity [8] between the set of genes in the signature and a subset of the genes that are known to be lower expressed when mouse embryonic fibroblasts are reprogrammed into induced pluripotent stem cells [9]. Because it is known [10] that a mesenchymal-epithelial transition (MET) is part of the reprogramming of mouse fibroblasts into stem cells, we reasoned that, conversely, the signature may correspond to some kind of EMT-related transition from a stem-like state to a fibroblast-like state used in early embryogenesis. Therefore, we hypothesized that many among the genes in the signature may be expressed by cancer stem cells (CSCs) passing through some type of EMT. Furthermore, because of the prominent presence of inhibin-A (*INHBA*) in the signature, we hypothesized that activin A (INHBA dimer) signaling may be responsible for the signature.

To test these two hypotheses and to identify which among the genes of the signature, if any, are expressed by the cancer cells, we used xenograft models implanting human cancer cell lines into NCR nude mice. Since the signature was clearly found in neuroblastoma [see Additional file 6], in which we already had successful experience of xenograft experimentation, we used a neuroblastoma cell line even though it is not strictly an epithelial tumor, as this could shed further light on the complex biological underlying mechanisms. Some of the implanted cancer cell lines were in their original form, some were engineered to express INHBA, and some were engineered to express the activin antagonist follistatin (FST). Each of the resulting growing tumors was harvested and profiled for gene expression twice using human and mouse microarrays separately. Our results validated the first hypothesis, but not the second: Most of the genes of the signature were found in human, but not mouse, cells, and their presence was independent of any transfections, indicating that activin signaling does not play a causal role.

**Figure 1:**
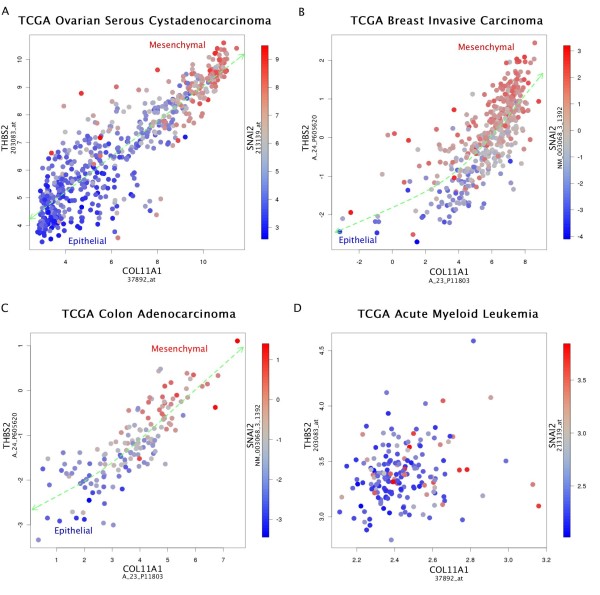
**Color-coded scatter plots for the coexpression of the EMT inducing transcription factor Slug (SNAI2) The scatter plots indicate the strong co-expression with the main signature genes COL11A1 and THBS2, as well as the continuity of the passing of cancer cells through a Slug-based EMT in solid tumors, and the total absence of the co-expression of these genes otherwise**. **A**, **B **and **C**, plots from three solid tumor datasets. **D**, plot from a leukemia dataset.

Specifically, our experiments confirmed that the genes of the signature, Slug being prominent among them, are co-expressed across samples to various degrees covering a continuous range of values including some samples with large expression of these genes. This result is consistent with the publicly available cancer gene expression datasets. Figure 1 shows that Slug is indeed co-expressed with the key genes *COL11A1 *and *THBS2 *in various cancer types, but not leukemia. The remarkable continuity of the dots shown in the scatter plots of Figure 1A-C suggests a dynamic and potentially reversible process as the human cancer cells pass through a mesenchymal transition process.

## Methods

### Tumor implantation

An inoculum of 106 NGP neuroblastoma cells containing FUW-Luciferase plasmid (kindly donated by Dr. Adolfo Ferrando) suspended in 0.1 mL of phosphate-buffered saline (PBS) was injected into the left kidney of 18 mice. The NGP cell line was originally obtained from Garrett Brodeur, Children's Hospital of Philadelphia. All cells were authenticated by short tandem repeat profiling analysis. Some of the implanted cells had previously been stably co-transfected with either FST-pReceiver-Lv105 or INHBA pReceiver-Lv105 (GeneCopoeia; Rockville, MD). Seven mice were implanted with INHBA-transfected NGP cells, six with FST and five with control NGP cells. All mice experiments and breeding were conducted according to protocols approved by the Columbia University IACUC.

### Harvesting of specimens

Mice were sacrificed when estimated tumor weight reached 1.5 g followed by collection of contralateral kidney and tumor. Tumor tissue was snap frozen for RNA isolation.

### Microarrays and probes preparation

HG-U133A 2.0 (human genome) and 430 A2.0 (mouse genome) Gene Chips (Affymetrix, Santa Clara, CA) were used to investigate gene expression in xenograft tumors. The cRNA probes were synthesized as recommended by Affymetrix, purified using RNeasy and fragmented according to the Affymetrix protocol, and 15 μg of biotinylated cRNA were hybridized to the microarrays. The samples were scanned with Affymetrix Gene Chip Scanner 3000.

### RNA isolation and semiquantitative reverse transcription-PCR

Total RNA was isolated from tumors using Qiagen miRNeasy mini kit (Qiagen, Germantown, MD) followed by reverse transcription using SuperScript First-Strand synthesis System for RT-PCR from Invitrogen according to manufacture recommendations (Carlsbad, CA, USA). Relative expression of human versus mouse COL11A1 (Hs00266273_m1, Ms00483387_m1) genes in tumor xenografts were examined by RT-PCR. Products were detected by Hot Start-IT Probe qPCR Master Mix from USB Affymetrix (Santa Clara, CA) according to manufacture instructions and analyzed by Stratagene Mx3005p real time PCR machine. After scanning, expression values for genes were determined using MxPro 410 (Agilent Technologies, Inc., Santa Clara, CA). Human HPRT, CYC, GAPD and mouse GAPD, ACTB, GUSB expression values were used to correct for sample variations in RT-PCR efficiency and errors in quantitation.

### Microarray dataset

The data set, corresponding to 18 tumors profiled separately with human and mouse microarrays, has been deposited in NCBI's Gene Expression Omnibus (GEO) and is available through accession number GSE34481. Data were RMA normalized using the Bioconductor open source software.

### Differential expression analysis

We regrouped the 18 samples, according to the expression level of human COL11A1, into seven samples with high or intermediate COL11A1 expression values, and eleven with low COL11A1 expression values. Based on this partition, we performed differential expression analysis using significance analysis of microarrays (SAM) [11], implemented in the Bioconductor package samr. We define the significantly differentially expressed gene as those having both a *Q *value less than 0.05 and a fold-change greater than 2.

## Results

**Figure 2:**
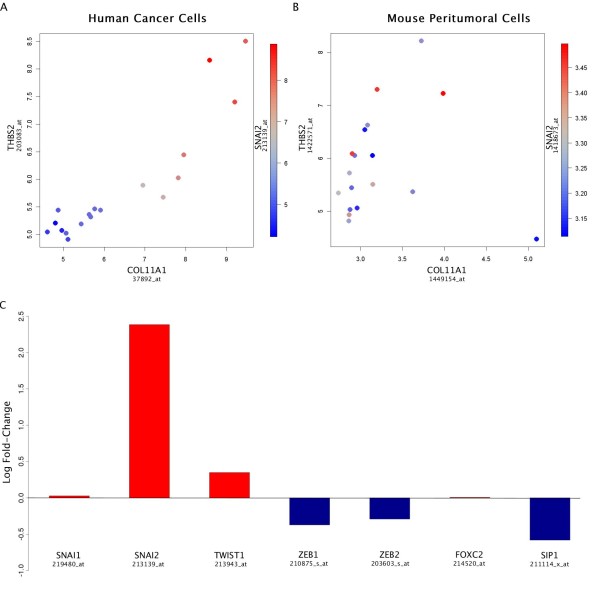
**Color-coded scatter plots in human and mouse of the 18 samples for Slug expression The expression of the EMT inducing transcription factor Slug (SNAI2) is shown in terms of the expression of the main signature genes COL11A1 and THBS2**. **A**, demonstration that this co-expression is present in the xenografted human cells. **B**, demonstration that this co-expression is absent in the peritumoral mouse cells. **C**, Bar diagram indicating that other EMT inducing transcription factors are not co-expressed.

**Figure 3:**
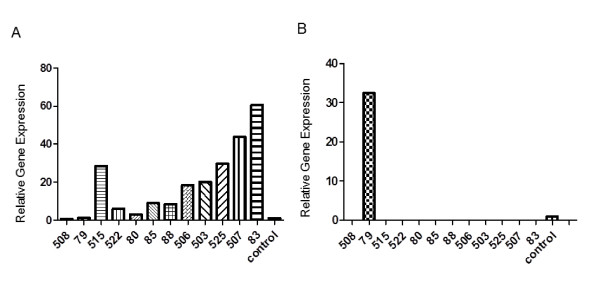
**Quantitative PCR Shown is the expression of human COL11A1 (**A**) or mouse COL11A1 (**B**) in RNA samples isolated from tumor NGP xenografts (number indicated)**. Expression of human COL11A1 increased in samples from left to right consistent with microarray results. Data were normalized to the expression of human HPRT. Mouse COL11A1 was not expressed in samples. Data was normalized to the expression of mouse Actb. Graphs depicted as relative levels of human (**A**) or mouse (**B**) control reference RNA.

We found very different expression levels (Figure 2) for most genes in human and mouse, suggesting that interspecies hybridization is minimal, as previously reported [12], which was confirmed with real-time PCR (see Materials and Methods and Figure 3). Using COL11A1 as a proxy, we ranked the 18 samples accordingly and investigated if most of the genes in Table 1 were co-expressed with *COL11A1*. We found that this was indeed the case in human cells only. For example, Figures 2A-B show color-coded scatter plots in human and mouse of the 18 samples for the expression of Slug (*SNAI2*) in terms of the expression of the main genes of the signature, *COL11A1 *and *THBS2 *(same as in the scatter plots of Figure 1 suggesting an identical biological process) demonstrating that this co-expression is clearly present in the human cells, but absent in the mouse cells. Specifically, seven samples had high or intermediate levels of co-expressed genes in the human cells, while the remaining 11 have relatively lower levels.

Based on this partition, we identified 398 significantly (both Q < 0.05 and FC > 2) up-regulated genes, 29 among which (*COL11A1*, *THBS2*, *COL5A2*, *COL5A1*, *VCAN*, *COL1A1*, *COL3A1*, *FN1*,*SULF1*, *FBN1*, *ASPN*, *SPARC*, *CTSK*, *MMP2*, *BGN*, *LUM*, *LOXL2*, *COL6A3*, *TIMP3*, *CDH11*, *SERPINF1*, *EDNRA*, *ACTA2*, *PDGFRB*, SNAI2, *LGALS1*, *GLT8D2*, *NID2*, *PRRX1*) belong to the set of 64 genes in Table 1 (*P *= 10^-27^), as well as *VIM *(vimentin). The presence in this list of *SNAI2 *(Slug), *ACTA2 *(α-SMA), *FN1 *(fibronectin), *VIM *(vimentin), together with many of the other EMT markers mentioned above, indicates that some human cancer cells underwent EMT. Other EMT-inducing transcription factors (Snail, Twist, ZEB1, ZEB2, SIP1, FOXC2) were not upregulated (Figure 2CC), while the upregulation of SNAI2 (Figure 2A) was very significant (*Q *< 3 × 10^-4 ^and FC = 5.22).

**Figure 4:**
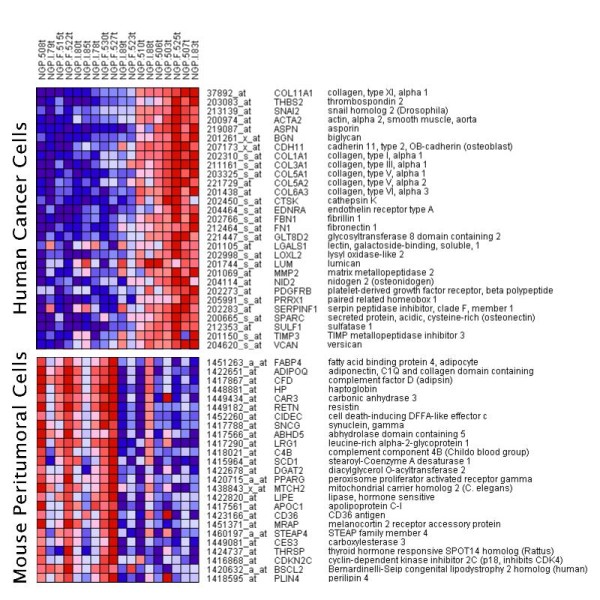
**Heat map combining human and mouse genes**. The 29 human genes include many EMT factors and were found to be significantly co-expressed in the cancer cells.

The heat map in Figure 4 shows the co-expression of the above 29 significantly upregulated genes. *INHBA*, the third prominent gene of the signature in addition to *COL11A1 *and *THBS2*, is not included in the list, because its expression was manipulated by the transfections with consistent results. Furthermore, the transfections of cancer cells with either INHBA (labeled I) or FST (labeled F) did not have any effect on the presence of the signature (the corresponding expression levels were consistent with the transfections and did not affect the expression of the other genes in the signature).

These same 29 genes are used in the heat maps shown in the Additional files, suggesting that the same signature may be expressed in all solid tumors, even in nonepithelial tumors, such as neuroblastoma and Ewing's sarcoma. Therefore, our results imply that the underlying mesenchymal transition process is more general than what EMT is presumed to be.

We next analyzed the mouse microarray data to identify genes correlated with the presence of the cancer EMT signature in the human cells. We found 32 significantly (both *Q *< 0.05 and FC > 2) downregulated mouse genes in the presence of the human cancer EMT signature. Among them, the top two genes with the highest fold change (12.3 and 11.8 respectively) were the adipocyte markers adiponectin (*ADIPOQ*) and adipsin (*CFD*). We observed that these genes were strongly co-regulated with many other adipocyte markers, including fatty acid binding protein 4 (*FABP4*) aka *aP2*. This cluster of adipocyte markers whose downregulation in the mouse cells is strongly associated with the upregulation of the EMT signature genes in human cells is also shown in the heat map of Figure 4. Many among these genes are known as adipocyte differentiation markers, and their downregulation is consistent with the finding that adipocytes are dedifferentiated as they encounter adjacent invading cancer cells in a "vicious cycle" [13] of a complex interaction facilitating cancer cell invasiveness. The observed downregulation of adipocyte markers in the adjacent mouse tissue, associated with the EMT-specific gene expression of the human cells provides a potential molecular control mechanism of microenvironmental contextual interactions in accordance with previously published results.

In summary, we have shown that a precise multi-cancer EMT signature present in numerous publicly available datasets was found in our xenograft experiments not to be expressed by the stromal cells, but instead by the human cancer cells themselves in vivo. Prior to our experiments, this was an open question. Indeed, similar signatures were usually referred to as "stromal," because they contain genes, such as a-SMA, that are typically found in stromal cells such as fibroblasts. Therefore, our results are consistent with the hypothesis that an EMT can convert cancer cells into mesenchymal, fibroblast-like cells that may well assume the duties of cancer-associated fibroblasts in some tumors [14]. Of additional significance is the knowledge of the precise composition of this particular Slug-based signature (as found both in our xenograft experiments as well as numerous cancer datasets) as it provides opportunities for identifying potential targets for inhibiting the underlying biological mechanism of mesenchymal transition of cancer cells.

## Discussion

The quality of "stemness" in cells appears to be closely interconnected with the ability to pass through transitions to and from mesenchymal characteristics. Indeed, EMT generates cells with properties of stem cells [15] and, conversely, MET is involved in the reprogramming of fibroblasts into stem cells [10]. Therefore, we speculate [8] that the cancer mesenchymal transition signature identified in this work may initiate from CSCs, which may even appear spontaneously [16]. It is also possible that the adipocytes of the stroma adjacent to the tumor are dedifferentiated into a mesenchymal stem cell-like state and, together with other mesenchymal stem cells derived from the bone marrow [17] interact with the invading fibroblastic cancer cells in a manner that reactivates a particular early embryonic developmental program.

Not all genes in Table 1 are upregulated in the human cells. For example, *PLAU *(urokinase plasminogen activator) is slightly upregulated only in mouse cells consistently with its assumed role of being secreted by reactive stromal cells as a pro-enzyme that activates itself and other proteases in the presence of cancer cells.

Another prominent gene of the signature observed in public databases that was not upregulated in the human xenografted cells is *MMP11 *(matrix metallopeptidase 11, aka stromelysin 3), in agreement with the hypothesis [13,18,19] that MMP11 is expressed in the adipocytes of the adjacent reactive stroma, indicating that the full version of the cancer mesenchymal transition signature may be stabilized by contextual microenvironmental signals when cancer cells encounter adipocytes triggering their dedifferentiation, apoptosis, or metabolism [13]. The expression in cancer cells of the adipocyte enhancer binding protein 1 (AEBP1), a prominent gene of the signature known to bind in the promoter region of the adipocyte fatty-acid protein FABP4 may play an important role in that respect, as may also the presence of oxidative stress and TNF signaling suggested by the presence of TNFAIP6 and C1QTNF3 in the signature.

The hypothesis that the mesenchymal transition signature is triggered by adipocytes is consistent with the facts [5] that in breast cancer the signature overexpression appears immediately upon reaching invasive stage I, while in ovarian cancer overexpression appears only when the tumor is already well into stage III as in omental metastasis, because ovarian cancer initially progresses by disseminating locally across mesothelial surfaces and probably carried by the physiological movement of peritoneal fluid to the peritoneum and omentum, a fatty structure [20].

The remarkable continuity of the signature (Figures 1 and 2) suggests that it may reflect a reversible process in which the mesenchymal markers in the cancer cells may appear and disappear depending on microenvironmental contextual signals. Its potential reversal is consistent with the possibility that passing through this mesenchymal transition at some point is a requirement for the initial stage of all metastases, even though the signature is significantly observed only in a subset of high-stage extracted samples at any given time. In other words, the lack of the signature in a particular extracted sample does not imply that cancer is not invasive, because the signature may have appeared earlier. However, the presence of the signature implies that the cancer is invasive; therefore a potential related biomarker product would have high selectivity, but not sensitivity.

## Conclusions

Many among the genes of the signature expressed by the human xenografted cells have previously been individually identified as associated with metastatic potential in cancer. Such associations can now largely be explained by the fact that these genes are expressed by the cancer cells themselves undergoing a mesenchymal transition. The known precise composition of the signature, particularly when combined with its separation in a species-specific manner in xenograft models, provides multiple and unique opportunities for understanding the underlying biological mechanisms and identifying diagnostic and prognostic markers as well as potential targets for metastasis-inhibiting therapeutics.

## Competing interests

The authors declare that they have no competing interests.

## Authors' contributions

JJK, DJY and DA designed the experiments. VR performed the genetic experiments. WYC performed the computational experiments. All authors contributed to the analysis and interpretation of data. DA drafted the initial version of the manuscript. All authors contributed to the formulation of the final manuscript, which they read and approved.

## Pre-publication history

The pre-publication history for this paper can be accessed here:

http://www.biomedcentral.com/1471-2407/11/529/prepub

## Supplementary Material

Additional file 1 **Heat map of breast cancer data set This file contains the heat map of the TCGA breast cancer data set for the genes of the mesenchymal transition signature**. Click here for file

Additional file 2 **Heat map of colon cancer data set This file contains the heat map of the TCGA colon cancer data set for the genes of the mesenchymal transition signature**. Click here for file

Additional file 3 **Heat map of Ewing's sarcoma data set This file contains the heat map of a Ewing's sarcoma data set (GEO accession number GSE12102) for the genes of the mesenchymal transition signature**. Click here for file

Additional file 4 **Heat map of lung cancer data set This file contains the heat map of the TCGA lung cancer data set for the genes of the mesenchymal transition signature**. Click here for file

Additional file 5 **Heat map of ovarian cancer data set This file contains the heat map of the TCGA ovarian cancer data set for the genes of the mesenchymal transition signature**. Click here for file

Additional file 6 **Heat map of neuroblastoma data set This file contains the heat map of a neuroblastoma data set (GEO accession number GSE3960) for the genes of the mesenchymal transition signature**. Click here for file

Additional file 7 **Heat map of leukemia data set This file contains the heat map of the TCGA leukemia data set for the genes of the mesenchymal transition signature**. Click here for file
