# Supplementary material for: Human cancer cells express Slug-based epithelial-mesenchymal transition gene expression signature obtained in vivo
Source: BMC Cancer. 2011 Dec 30;11:529. doi: 10.1186/1471-2407-11-529 (PMC3268117; doi:10.1186/1471-2407-11-529)
Supplement: Additional file 2 — Heat map of colon cancer data set This file contains the heat map of the TCGA colon cancer data set for the genes of the mesenchymal transition signature. [file 1471-2407-11-529-S2.PDF]

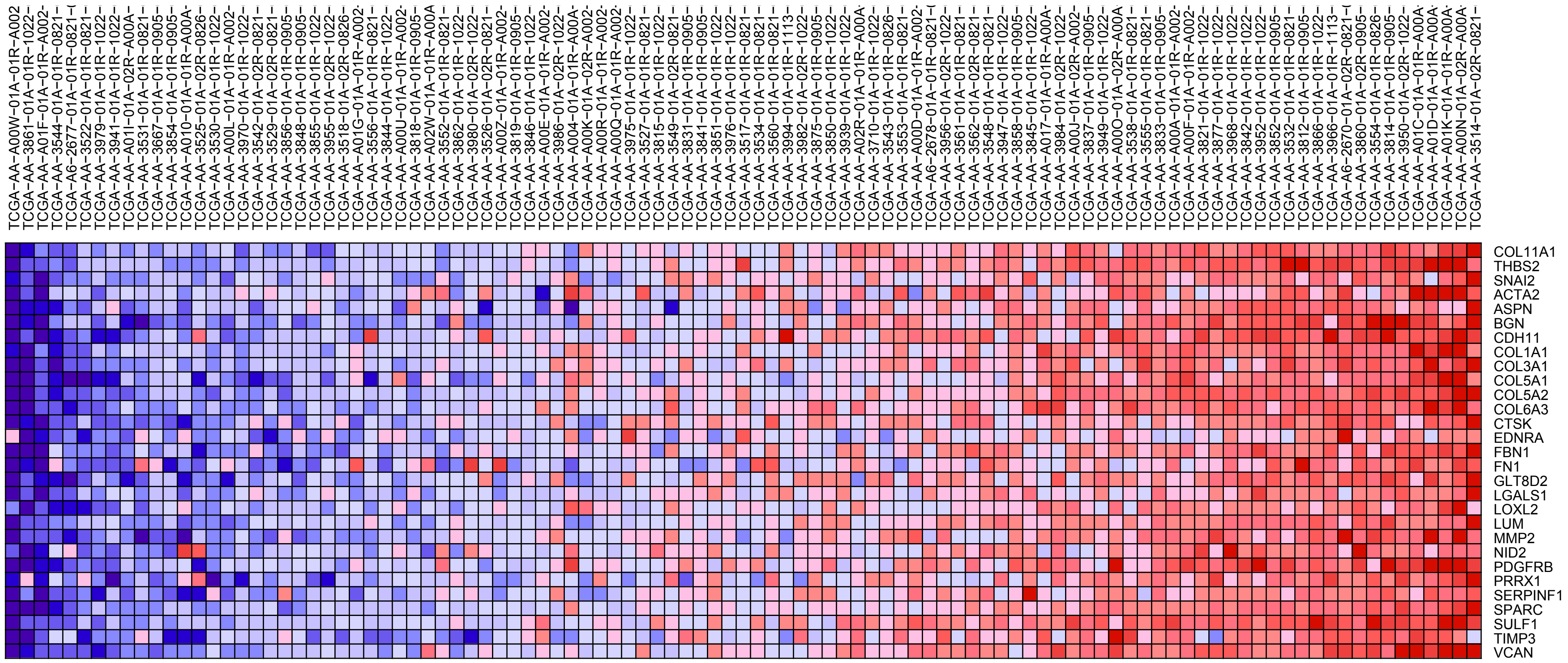
TCGA-AA-A00W-01A-01R-A002
TCGA-AA-3861-01A-01R-1022
TCGA-AA-A01F-01A-01R-A002
TCGA-AA-3544-01A-01R-0821
TCGA-A6-2677-01A-01R-0821-(
TCGA-AA-3522-01A-01R-0821
TCGA-AA-3979-01A-01R-1022
TCGA-AA-3941-01A-01R-1022
TCGA-AA-A01I-01A-02R-A00A
TCGA-AA-3531-01A-01R-0821
TCGA-AA-3667-01A-01R-0905
TCGA-AA-3854-01A-01R-0905
TCGA-AA-A010-01A-01R-A00A
TCGA-AA-3525-01A-02R-0826
TCGA-AA-3530-01A-01R-1022
TCGA-AA-A00L-01A-01R-A002
TCGA-AA-3970-01A-01R-1022
TCGA-AA-3542-01A-02R-0821
TCGA-AA-3529-01A-02R-0821
TCGA-AA-3856-01A-01R-0905
TCGA-AA-3848-01A-01R-1022
TCGA-AA-3855-01A-01R-1022
TCGA-AA-3955-01A-02R-1022
TCGA-AA-3518-01A-02R-0826
TCGA-AA-A01G-01A-01R-A002
TCGA-AA-3556-01A-01R-0821
TCGA-AA-3844-01A-01R-1022
TCGA-AA-A00U-01A-01R-A002
TCGA-AA-3818-01A-01R-0905
TCGA-AA-A02W-01A-01R-A00A
TCGA-AA-3552-01A-01R-0821
TCGA-AA-3862-01A-01R-1022
TCGA-AA-3980-01A-02R-1022
TCGA-AA-3526-01A-02R-0821
TCGA-AA-A00Z-01A-01R-A002
TCGA-AA-3819-01A-01R-0905
TCGA-AA-3846-01A-01R-1022
TCGA-AA-A00E-01A-01R-A002
TCGA-AA-3986-01A-02R-1022
TCGA-AA-A004-01A-01R-A00A
TCGA-AA-A00K-01A-02R-A002
TCGA-AA-A00R-01A-02R-A002
TCGA-AA-A00Q-01A-01R-A002
TCGA-AA-3975-01A-01R-1022
TCGA-AA-3527-01A-01R-0821
TCGA-AA-3815-01A-01R-1022
TCGA-AA-3549-01A-02R-0821
TCGA-AA-3831-01A-01R-0905
TCGA-AA-3841-01A-01R-0905
TCGA-AA-3851-01A-01R-1022
TCGA-AA-3976-01A-01R-1022
TCGA-AA-3517-01A-01R-0821
TCGA-AA-3534-01A-01R-0821
TCGA-AA-3560-01A-01R-0821
TCGA-AA-3994-01A-01R-1113
TCGA-AA-3982-01A-02R-1022
TCGA-AA-3875-01A-02R-0905
TCGA-AA-3850-01A-01R-1022
TCGA-AA-3939-01A-01R-1022
TCGA-AA-A02R-01A-01R-A00A
TCGA-AA-3710-01A-01R-0826
TCGA-AA-3543-01A-01R-0821
TCGA-AA-3553-01A-01R-0821
TCGA-AA-A00D-01A-01R-A002
TCGA-A6-2678-01A-01R-0821-(
TCGA-AA-3956-01A-02R-1022
TCGA-AA-3561-01A-01R-0821
TCGA-AA-3562-01A-02R-0821
TCGA-AA-3548-01A-01R-0821
TCGA-AA-3947-01A-01R-1022
TCGA-AA-3858-01A-01R-0905
TCGA-AA-3845-01A-01R-1022
TCGA-AA-A017-01A-01R-A00A
TCGA-AA-3984-01A-02R-1022
TCGA-AA-A00J-01A-02R-A002
TCGA-AA-3837-01A-01R-0905
TCGA-AA-3949-01A-01R-1022
TCGA-AA-A00O-01A-02R-A00A
TCGA-AA-3538-01A-01R-0821
TCGA-AA-3555-01A-01R-0821
TCGA-AA-3833-01A-01R-0905
TCGA-AA-A00A-01A-01R-A002
TCGA-AA-A00F-01A-01R-A002
TCGA-AA-3821-01A-01R-1022
TCGA-AA-3877-01A-01R-1022
TCGA-AA-3968-01A-01R-1022
TCGA-AA-3842-01A-01R-1022
TCGA-AA-3952-01A-01R-1022
TCGA-AA-3852-01A-01R-0905
TCGA-AA-3532-01A-01R-0821
TCGA-AA-3812-01A-01R-0905
TCGA-AA-3866-01A-01R-1022
TCGA-AA-3966-01A-01R-1113
TCGA-A6-2670-01A-02R-0821-(
TCGA-AA-3860-01A-02R-0905
TCGA-AA-3554-01A-01R-0826
TCGA-AA-3814-01A-01R-0905
TCGA-AA-3950-01A-02R-1022
TCGA-AA-A01C-01A-01R-A00A
TCGA-AA-A01D-01A-01R-A00A
TCGA-AA-A01K-01A-01R-A00A
TCGA-AA-A00N-01A-02R-A00A
TCGA-AA-3514-01A-02R-0821
COL11A1
THBS2
SNAI2
ACTA2
ASPN
BGN
CDH11
COL1A1
COL3A1
COL5A1
COL5A2
COL6A3
CTSK
EDNRA
FBN1
FN1
GLT8D2
LGALS1
LOXL2
LUM
MMP2
NID2
PDGFRB
PRRX1
SERPINF1
SPARC
SULF1
TIMP3
VCAN
