# Supplementary figures and images for: Human cancer cells express Slug-based epithelial-mesenchymal transition gene expression signature obtained in vivo
Source: BMC Cancer. 2011 Dec 30;11:529. doi: 10.1186/1471-2407-11-529 (PMC3268117; doi:10.1186/1471-2407-11-529)

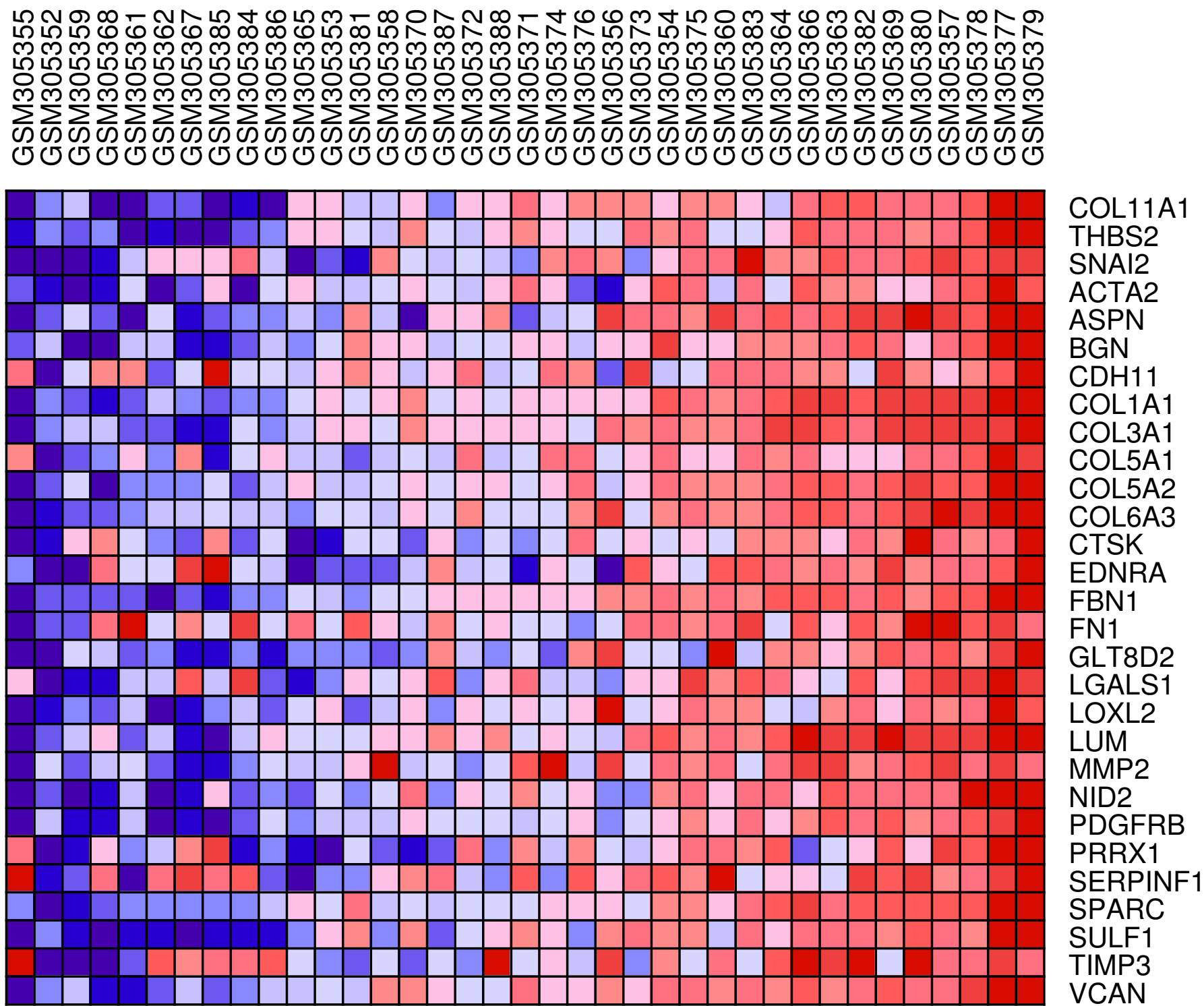

Supplement: Additional file 3 — Heat map of Ewing's sarcoma data set This file contains the heat map of a Ewing's sarcoma data set (GEO accession number GSE12102) for the genes of the mesenchymal transition signature. [file 1471-2407-11-529-S3.PDF]

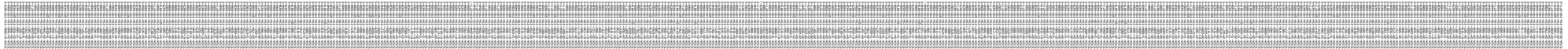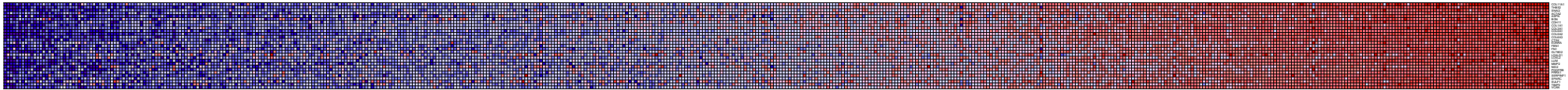

Supplement: Additional file 5 — Heat map of ovarian cancer data set This file contains the heat map of the TCGA ovarian cancer data set for the genes of the mesenchymal transition signature. [file 1471-2407-11-529-S5.PDF]
