# Supplementary material for: Human cancer cells express Slug-based epithelial-mesenchymal transition gene expression signature obtained in vivo
Source: BMC Cancer. 2011 Dec 30;11:529. doi: 10.1186/1471-2407-11-529 (PMC3268117; doi:10.1186/1471-2407-11-529)
Supplement: Additional file 4 — Heat map of lung cancer data set This file contains the heat map of the TCGA lung cancer data set for the genes of the mesenchymal transition signature. [file 1471-2407-11-529-S4.PDF]

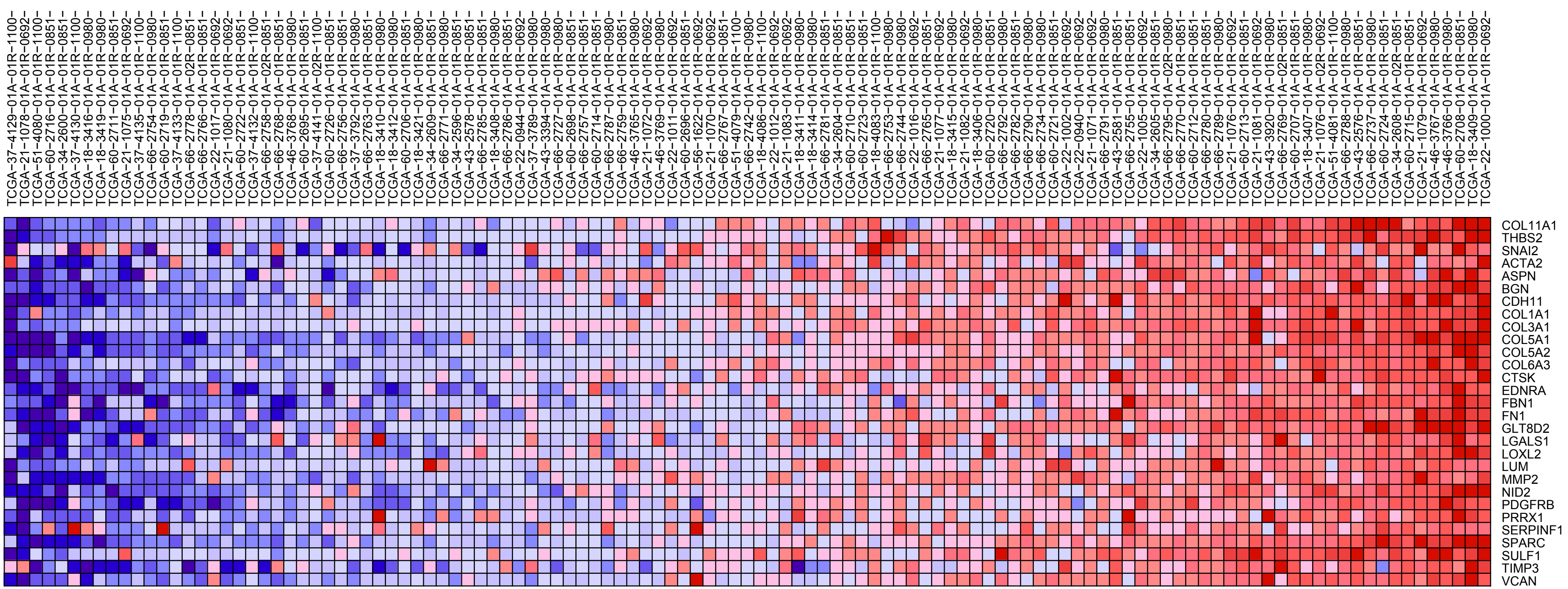
TCGA-37-4129-01A-01R-1100
TCGA-21-1078-01A-01R-0692
TCGA-51-4080-01A-01R-1100
TCGA-60-2716-01A-01R-0851
TCGA-34-2600-01A-01R-0851
TCGA-37-4130-01A-01R-1100
TCGA-18-3416-01A-01R-0980
TCGA-18-3419-01A-01R-0980
TCGA-60-2711-01A-01R-0851
TCGA-21-1075-01A-01R-0692
TCGA-37-4135-01A-01R-1100
TCGA-66-2754-01A-01R-0980
TCGA-60-2719-01A-01R-0851
TCGA-37-4133-01A-01R-1100
TCGA-66-2778-01A-02R-0851
TCGA-66-2766-01A-01R-0851
TCGA-22-1017-01A-01R-0692
TCGA-21-1080-01A-01R-0692
TCGA-60-2722-01A-01R-0851
TCGA-37-4132-01A-01R-1100
TCGA-66-2758-01A-02R-0851
TCGA-66-2768-01A-01R-0851
TCGA-46-3768-01A-01R-0980
TCGA-60-2695-01A-01R-0851
TCGA-37-4141-01A-02R-1100
TCGA-60-2726-01A-01R-0851
TCGA-66-2756-01A-01R-0851
TCGA-37-3792-01A-01R-0980
TCGA-66-2763-01A-01R-0851
TCGA-18-3410-01A-01R-0980
TCGA-18-3412-01A-01R-0980
TCGA-60-2706-01A-01R-0851
TCGA-18-3421-01A-01R-0980
TCGA-34-2609-01A-01R-0851
TCGA-66-2771-01A-01R-0980
TCGA-34-2596-01A-01R-0851
TCGA-43-2578-01A-01R-0851
TCGA-66-2785-01A-01R-0851
TCGA-18-3408-01A-01R-0980
TCGA-66-2786-01A-01R-0851
TCGA-22-0944-01A-01R-0692
TCGA-37-3789-01A-01R-0980
TCGA-43-3394-01A-01R-0980
TCGA-60-2727-01A-01R-0851
TCGA-60-2698-01A-01R-0851
TCGA-66-2757-01A-01R-0851
TCGA-60-2714-01A-01R-0851
TCGA-66-2787-01A-01R-0980
TCGA-66-2759-01A-01R-0851
TCGA-46-3765-01A-01R-0980
TCGA-21-1072-01A-01R-0692
TCGA-46-3769-01A-01R-0980
TCGA-22-1011-01A-01R-0692
TCGA-60-2696-01A-01R-0851
TCGA-56-1622-01A-01R-0692
TCGA-21-1070-01A-01R-0692
TCGA-66-2767-01A-01R-0851
TCGA-51-4079-01A-01R-1100
TCGA-66-2742-01A-01R-0980
TCGA-18-4086-01A-01R-1100
TCGA-22-1012-01A-01R-0692
TCGA-21-1083-01A-01R-0692
TCGA-18-3411-01A-01R-0980
TCGA-66-2781-01A-01R-0851
TCGA-34-2604-01A-01R-0851
TCGA-60-2710-01A-01R-0851
TCGA-60-2723-01A-01R-0851
TCGA-18-4083-01A-01R-1100
TCGA-66-2753-01A-01R-0980
TCGA-66-2744-01A-01R-0980
TCGA-22-1016-01A-01R-0692
TCGA-66-2765-01A-01R-0851
TCGA-21-1077-01A-01R-0692
TCGA-18-3415-01A-01R-0980
TCGA-21-1082-01A-01R-0692
TCGA-18-3406-01A-01R-0980
TCGA-60-2720-01A-01R-0851
TCGA-66-2792-01A-01R-0980
TCGA-66-2782-01A-01R-0851
TCGA-66-2790-01A-01R-0980
TCGA-66-2734-01A-01R-0980
TCGA-60-2721-01A-01R-0851
TCGA-22-1002-01A-01R-0692
TCGA-22-0940-01A-01R-0692
TCGA-21-1071-01A-01R-0692
TCGA-66-2791-01A-01R-0980
TCGA-43-2581-01A-01R-0851
TCGA-66-2755-01A-01R-0851
TCGA-22-1005-01A-01R-0692
TCGA-34-2605-01A-01R-0851
TCGA-66-2795-01A-02R-0980
TCGA-66-2770-01A-01R-0851
TCGA-60-2712-01A-01R-0851
TCGA-66-2780-01A-01R-0851
TCGA-66-2789-01A-01R-0980
TCGA-21-1076-01A-01R-0692
TCGA-60-2713-01A-01R-0851
TCGA-21-1081-01A-01R-0692
TCGA-43-3920-01A-01R-0980
TCGA-66-2769-01A-02R-0851
TCGA-60-2707-01A-01R-0851
TCGA-18-3407-01A-01R-0980
TCGA-21-1076-01A-02R-0692
TCGA-51-4081-01A-01R-1100
TCGA-66-2788-01A-01R-0980
TCGA-43-2576-01A-01R-0851
TCGA-66-2737-01A-01R-0980
TCGA-60-2724-01A-01R-0851
TCGA-34-2608-01A-02R-0851
TCGA-60-2715-01A-01R-0851
TCGA-21-1079-01A-01R-0692
TCGA-46-3767-01A-01R-0980
TCGA-46-3766-01A-01R-0851
TCGA-60-2708-01A-01R-0851
TCGA-18-3409-01A-01R-0980
TCGA-22-1000-01A-01R-0692
COL11A1
THBS2
SNAI2
ACTA2
ASPN
BGN
CDH11
COL1A1
COL3A1
COL5A1
COL5A2
COL6A3
CTSK
EDNRA
FBN1
FN1
GLT8D2
LGALS1
LOXL2
LUM
MMP2
NID2
PDGFRB
PRRX1
SERPINF1
SPARC
SULF1
TIMP3
VCAN
