# Supplementary material for: Human cancer cells express Slug-based epithelial-mesenchymal transition gene expression signature obtained in vivo
Source: BMC Cancer. 2011 Dec 30;11:529. doi: 10.1186/1471-2407-11-529 (PMC3268117; doi:10.1186/1471-2407-11-529)
Supplement: Additional file 6 — Heat map of neuroblastoma data set This file contains the heat map of a neuroblastoma data set (GEO accession number GSE3960) for the genes of the mesenchymal transition signature. [file 1471-2407-11-529-S6.PDF]

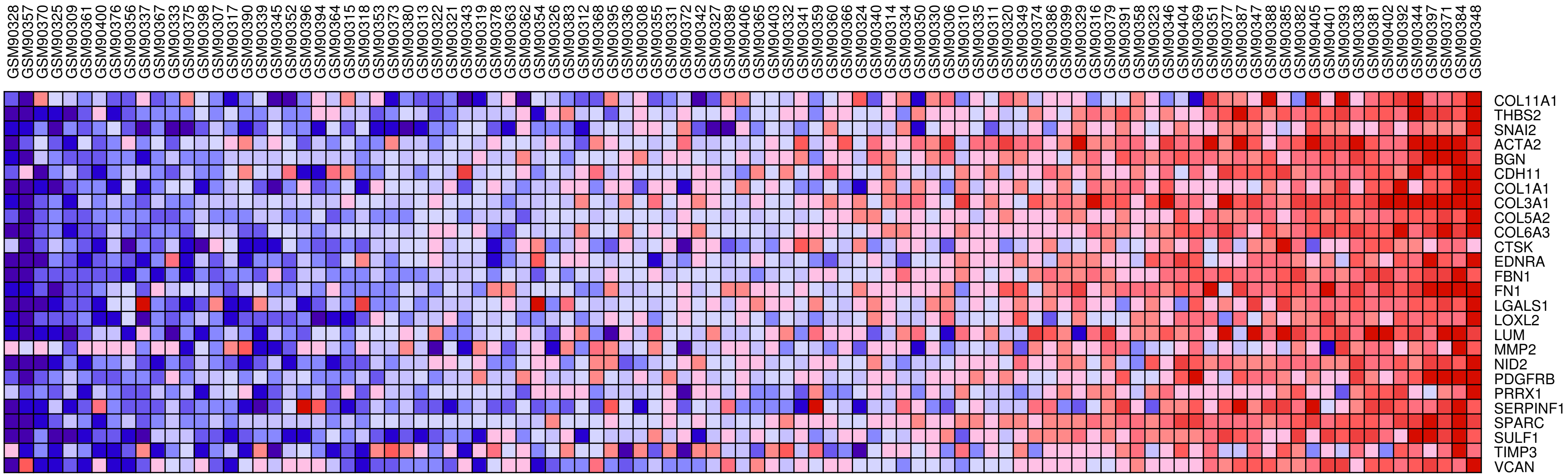
GSM90328
GSM90357
GSM90370
GSM90325
GSM90309
GSM90361
GSM90400
GSM90376
GSM90356
GSM90337
GSM90367
GSM90333
GSM90375
GSM90398
GSM90307
GSM90317
GSM90390
GSM90339
GSM90345
GSM90352
GSM90396
GSM90394
GSM90364
GSM90315
GSM90318
GSM90353
GSM90373
GSM90380
GSM90313
GSM90322
GSM90321
GSM90343
GSM90319
GSM90378
GSM90363
GSM90362
GSM90354
GSM90326
GSM90383
GSM90312
GSM90368
GSM90395
GSM90336
GSM90308
GSM90355
GSM90331
GSM90372
GSM90342
GSM90327
GSM90389
GSM90406
GSM90365
GSM90403
GSM90332
GSM90341
GSM90359
GSM90360
GSM90366
GSM90324
GSM90340
GSM90314
GSM90334
GSM90350
GSM90330
GSM90306
GSM90310
GSM90335
GSM90311
GSM90320
GSM90349
GSM90374
GSM90386
GSM90399
GSM90329
GSM90316
GSM90379
GSM90391
GSM90358
GSM90323
GSM90346
GSM90404
GSM90369
GSM90351
GSM90377
GSM90387
GSM90347
GSM90388
GSM90385
GSM90382
GSM90405
GSM90401
GSM90393
GSM90338
GSM90381
GSM90402
GSM90392
GSM90344
GSM90397
GSM90371
GSM90384
GSM90348
COL11A1
THBS2
SNAI2
ACTA2
BGN
CDH11
COL1A1
COL3A1
COL5A2
COL6A3
CTSK
EDNRA
FBN1
FN1
LGALS1
LOXL2
LUM
MMP2
NID2
PDGFRB
PRRX1
SERPINF1
SPARC
SULF1
TIMP3
VCAN
