# Supplementary material for: Human cancer cells express Slug-based epithelial-mesenchymal transition gene expression signature obtained in vivo
Source: BMC Cancer. 2011 Dec 30;11:529. doi: 10.1186/1471-2407-11-529 (PMC3268117; doi:10.1186/1471-2407-11-529)
Supplement: Additional file 7 — Heat map of leukemia data set This file contains the heat map of the TCGA leukemia data set for the genes of the mesenchymal transition signature. [file 1471-2407-11-529-S7.PDF]

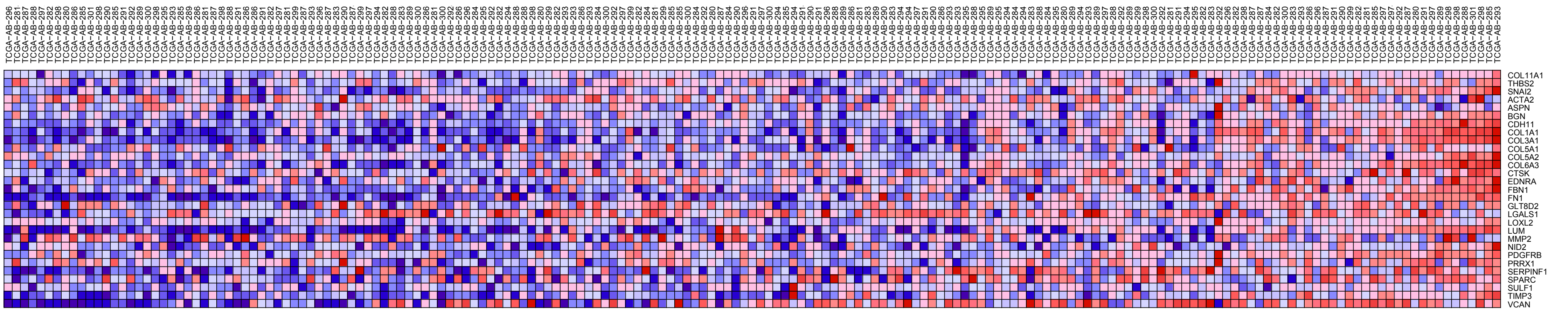
COL11A1
THBS2
SNAI2
ACTA2
ASPN
BGN
CDH11
COL1A1
COL3A1
COL5A1
COL5A2
COL6A3
CTSK
EDNRA
FBN1
FN1
GLT8D2
LGALS1
LOXL2
LUM
MMP2
NID2
PDGFRB
PRRX1
SERPINF1
SPARC
SULF1
TIMP3
VCAN
